# Bridge Health Monitoring Using Strain Data and High-Fidelity Finite Element Analysis

**DOI:** 10.3390/s22145172

**Published:** 2022-07-10

**Authors:** Behzad Ghahremani, Alireza Enshaeian, Piervincenzo Rizzo

**Affiliations:** Laboratory for Nondestructive Evaluation and Structural Health Monitoring Studies, Department of Civil and Environmental Engineering, University of Pittsburgh, 3700 O’Hara Street, Pittsburgh, PA 15261, USA; beg66@pitt.edu (B.G.); ale69@pitt.edu (A.E.)

**Keywords:** structural health monitoring, finite element modeling, bridge monitoring, strain sensors

## Abstract

This article presented a physics-based structural health monitoring (SHM) approach applied to a pretensioned adjacent concrete box beams bridge in order to predict the deformations associated with the presence of transient loads. A detailed finite element model was generated using ANSYS software to create an accurate model of the bridge. The presence of concentrated loads on the deck at different locations was simulated, and a static analysis was performed to quantify the deformations induced by the loads. Such deformations were then compared to the strains recorded by an array of wireless strain gauges during a controlled truckload test performed by an independent third party. The test consisted of twenty low-speed crossings at controlled distances from the bridge parapets using a truck with a certified load. The array was part of a SHM system that consisted of 30 wireless strain gauges. The results of the comparative analysis showed that the proposed physics-based monitoring is capable of identifying sensor-related faults and of determining the load distributions across the box beams. In addition, the data relative to near two-years monitoring were presented and showed the reliability of the SHM system as well as the challenges associated with environmental effects on the strain reading. An ongoing study is determining the ability of the proposed physics-based monitoring at estimating the variation of strain under simulated damage scenarios.

## 1. Introduction

Some bridges worldwide are either in poor condition or are operating beyond their designed life. In addition, the growth of urban areas increases the tonnage of commodities and the volume of vehicular and public transportation traffic moved daily [1]. According to 2019 statistics [2], there are 616,096 highway bridges in the USA, 46% of which are rated in good condition, 46.4% are rated fair, and 7.6% are rated poor [3]. Other statistics identified 54,259 bridges as “poor condition”. The fact that traditional periodic inspections may miss the onset of new damage or the trigger of critical issues between two inspections can be overcome with the implementation of cost-effective structural health monitoring (SHM) strategies, which evolve the maintenance paradigm from “time-based” nondestructive evaluation to permanent-based where sensors monitor structures 24/7 to flag, locate, and quantify damage as it happens. SHM systems make use of sensors to measure physical characteristics such as strain, acceleration, and temperature, just to mention a few, while dedicated hardware/software elaborates the set of time series streamed from the sensors. SHM methods can be data-driven or physics-based [4]. Data-driven methods identify methods that use damage-sensitive features extracted from field data [5]. These features can then be coupled to an automatic classification algorithm able to detect the presence of defects (unsupervised learning class) or to determine the presence, the size, and the location of defects (supervised learning class). The first class offers the fundamental advantage that the information on the damage conditions do not need to be known already, contrarily to the supervised learning class. The unsupervised approach is particularly useful in the case of complex or expensive structures where it is difficult to simulate damage prior to the deployment and the use of the monitoring system [6]. Data-driven methods do not require a physics model of the structure. Examples of data-driven approaches are the studies presented in refs. [7,8,9,10,11,12,13,14,15,16]. Liu et al. [7] applied neural network and multi-sensor feature fusion theory to classify the recorded sensor’s data. Gu et al. [8] used a two-step damage-detection technique to eliminate the effect of temperature in natural frequencies changing using artificial neural networks. Xu et al. [9] utilized a restricted Boltzmann machine in order to identify cracks in steel box girders of bridges. Azimi and Pekcan [10] studied the application of convolutional neural networks in damage detection of realistic large-scale systems. Ghahremani et al. [11] investigated damage detection in a three-story model using convolutional neural network and recorded acceleration signals. Bao et al. [12] proposed a two-step procedure to identify the anomalies caused by structural damage using a deep neural network. Shang et al. [13] developed a deep convolutional denoising autoencoder network to extract damage features from field measurements of undamaged structures in presence of noise and uncertainties. Abdeljaber et al. [14] investigated the application of 1D convolutional neural network in data fusion and damage-sensitive feature extraction from raw acceleration signals in order to detect and localize the damage in large-scale structures. Alamdari et al. [15] used a modified k-means clustering algorithm to identify potential structural damage in jack arches of Sydney Harbour Bridge. Fallahian et al. [16] presented a framework based on the combination of couple sparse coding and a deep neural network to assess damage in a noisy environment. Data-driven SHM methods applied to bridges present some challenges. As a matter of fact, unsupervised learning algorithms require a sufficiently large number of data that include all possible baseline configurations, whereas supervised methods require data from damaged configurations, which cannot always be realistic in practice [17,18,19]. In another group of data-driven methods, the long-term monitoring data were analyzed under ambient effects such as temperature variations to detect the anomalies in the recorded response of bridges. Under this framework, Wu et al. [20], Huang et al. [21], Xia et al. [22], and Wei et al. [23] used strain data, and Yang et al. used deflection data [24] and acceleration data [25].

Typically, physics-based methods rely on finite element (FE) models that may or may not be updated with field data [26,27,28,29,30,31,32,33,34,35,36,37,38,39]. Yu et al. [26] created a FE model of the Aizhai suspension bridge to detect damage and the model was updated using the information collected from 112 sensors of various types. Schlune et al. [27] developed a FE model for a single-arch bridge, the new Svinesund Bridge, to evaluate the health status of the bridge using static and dynamic measurements. Yang et al. [28] utilized 3D terrestrial laser scanning to measure the static response for FE model updating. He et al. [29] generated a three-dimensional FE model of the Nanjing Yangtze River Bridge with ANSYS. They used stress–time histories to validate the updated FE model and the results showed a good agreement between the calculated data and the tested data. Giagopoulos et al. [30] proposed a framework for fatigue damage identification using operational experimental measurements and a high-fidelity FE model. Ghahremani et al. [31] applied an evolutionary optimization algorithm to update numerical FE models of truss and frame structures. Schommer et al. [32] investigated SHM on a pre-stressed concrete beam based on the created FE model and static and dynamic responses. They also considered the effect of temperature on the recorded response of the concrete beam. Zanjani Zadeh and Patniak [33] developed a 3D FE model of a composite steel stringer supported reinforcement concrete deck highway bridge. They used the measured data during moving truck load tests to update the model properties. Gatti [34] compared the structural responses induced by static and dynamic load tests with the predicted ones from a FE model to monitor a pre-stressed reinforced concrete bridge built in the late 1960s. Cheng et al. [35] established a FE model for a 131 m large transmission tower to perform SHM. By manually tuning and updating the model, they could obtain a realistic model that can reproduce the measured experimental dynamic characteristics. Shun and Hong-ping [36] utilized substructure-based FE model updating to identify damage in a high-rise building model. Duan et al. [37] developed a detailed 3D FE model of the Tsing Ma Bridge, a cable suspension bridge, to compute and compare the stress/strain states of important bridge members. More research within the physics-based methods area can be found in [38,39].

In the study presented in this article, a high-fidelity finite element model of a two-span pretensioned concrete box beams bridge was created using ANSYS 2020 R2 software. Every single component of the bridge was included in the model and a static analysis was conducted by considering the presence of a truck on the bridge. The deformations predicted numerically were compared to the empirical data obtained by an independent third party during a truck-controlled loading test using an array of wireless strain gauges bolted to the bridge. The empirical data were collected from a repository and processed using a simple approach to remove any bias generated by the temperature and any offset that may have been occurred at the time of sensor installation. The processing approach was then expanded to analyze the data collected during a two-year timeframe to identify the onset of critical issues. These long-term data were processed using simple but effective procedures in order to extract relevant information about the structure.

The paper is organized as follows. The next section provides a brief description of the bridge and the FE model. Section 3 describes the SHM system installed by the third party and the protocol of the truckload test. Section 4 summarizes the results of this test whereas Section 5 discusses the simulations performed with the model. Section 6 presents the main findings of the long-term monitoring. Section 7 ends the article with a few concluding remarks.

## 2. The Bridge and the Finite Element Modeling

**Figure 1:**
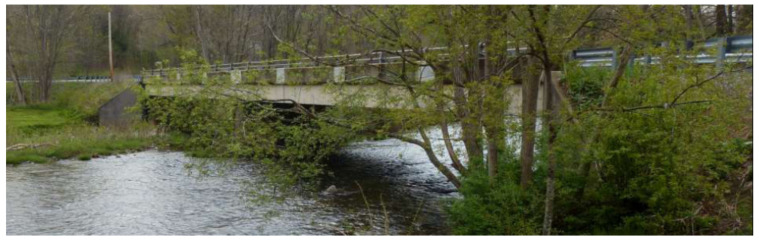
Upstream view of the 55-3014-0050-0509 bridge (Somerset bridge). Figure adapted from PennDOT bridge inspection 2014.

**Figure 2:**
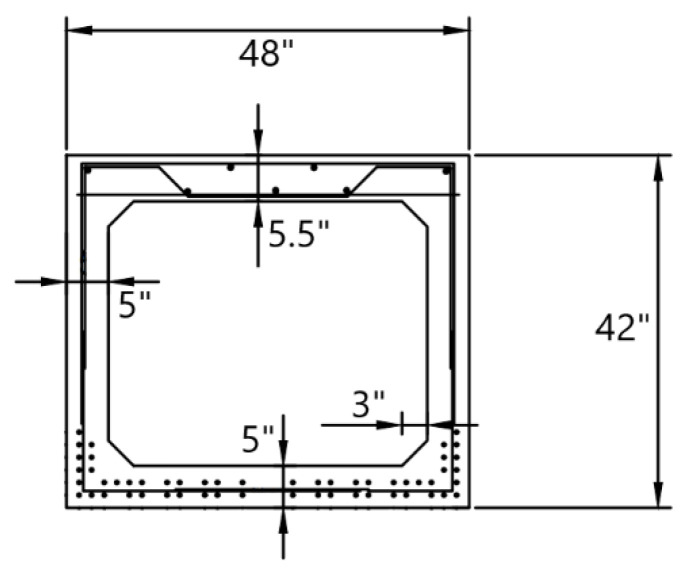
Cross section of the concrete box beams.

**Figure 3:**
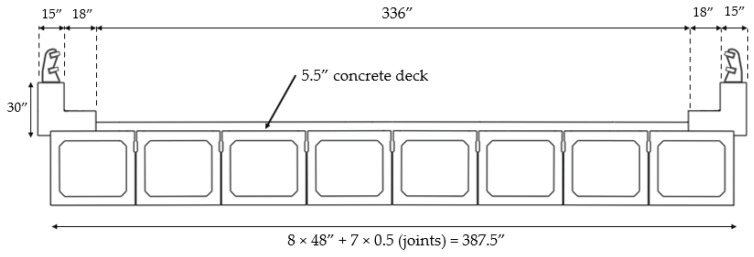
Cross section of the bridge.

The two spans BMS ID 55-3014-0050-0509 bridge (Figure 1) was built in 1959 and consists of pretensioned adjacent concrete box beams. Each span is 992.13 in long and is made of eight concrete box beams with a skew angle of 30°. The contact of the beams with the abutments and middle pier are “expandable” and “fixed”, respectively. There is no connection between the girders of the first and second spans. However, the girders of each span are laterally connected with shear keys. The cross-section of each box is shown in Figure 2. The reinforcement consists of 54 grade 250, 3/8-in diameter, seven-wire strands. The original concrete deck is 5.5 in thick. The cross-section of the bridge is presented in Figure 3. The material properties were extracted from the original drawings of the bridge; 5000 psi, 4000 psi, and 3500 psi concrete were used for beams, deck, and parapets, respectively. ASTM A615 grade 40 steel was used for the stirrups of the beams and grade 60 steel was used for rebars of the deck. Grade 250 steel was considered for strands of the beams.

**Figure 4:**
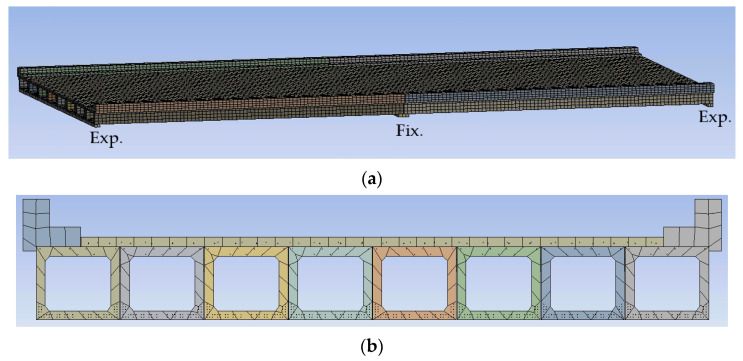
Snapshots from the FE model (**a**) overall look; (**b**) cross section.

The digital twin of the bridge was created using ANSYS 2020 R2 software (Figure 4). The beams, deck, and parapets were modeled as solid bodies and the strands, rebars, and stirrups were modeled as line bodies. Three-dimensional 20 node solid elements were considered for the analysis of the solid bodies, whereas REINF264 elements were used for the analysis of the line bodies. The overall model contained 83,891 elements. The mesh size was adjusted manually until a good trade-off between accuracy and computational time was found.

## 3. Sensor Installation and Load Tests

The SHM sensing system consisted of 30 bolted-type wireless strain gauges. Based on the third-party independent Vendor’s datasheet, the sensors were 1.35 in × 3.0 in × 0.6 in and about 90 g weight, with a working temperature range of −40 to +150 °F (−40 to +65 °C). Each strain gauge was also equipped with an embedded thermocouple. One sample was collected every 10 or 20 s for most of the recorded datapoints. However, the temperature was recorded every 6 min. Strain gauges have a resolution of 3.6 microstrain and cover a full range of ±4000 microstrains.

**Figure 5:**
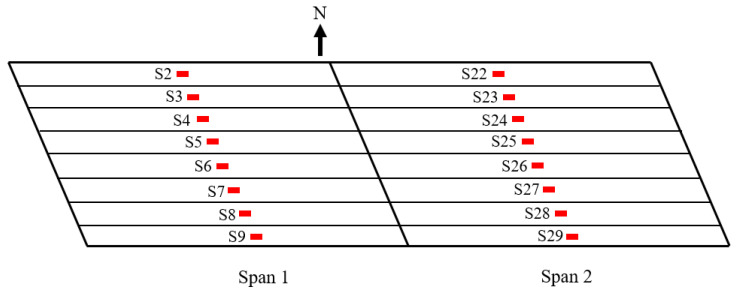
Location of the installed sensors.

Sixteen sensors were bolted at the bottom midpoint (50% length) of each floor beam of span 1 and span 2 as schematized in Figure 5. These sensors are labeled as S02–S09 and S22–S29. Four gauges were bolted at the midpoint of the outer face of the side girders (i.e., the girders that S2, S9, S22, and S29 are installed on). Span 1 was also instrumented with 10 sensors close to the center pier (97% length), two of which on the outside walls.

**Figure 6:**
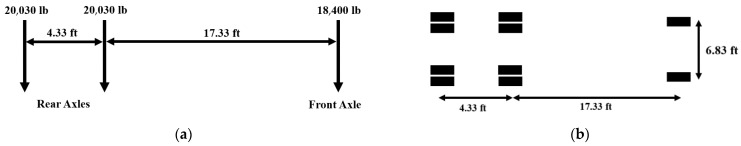
Details of the configuration and dimension of the test truck. (**a**) Side view; (**b**) top view.

The live load test was performed with a certified 58,460 lb truck. The steer axle was 18,400 lb whereas the drive ‘tandem’ axles were 40,060 lb (Figure 6). Twenty crossings at 5 mph were completed at a known lateral distance from the north parapet. The distances were 1 ft through 19 ft at 2 ft steps. Two crosses per distance were performed. As such, tests 1 and 2 were identical with the truck wheel 1 ft away from the north wall.

As each span can be considered as a simply supported beam, the maximum deflection and maximum strain/stress occurred when the truck was at midspan. Accordingly, to compare the measured maximum strains with the predicted ones, six point-loads were applied on the deck to simulate the weight of wheels and axles as shown in Figure 6b. As the truck moved slowly, its loading can be considered static and therefore any dynamic effect on the bridge was not studied here.

## 4. Field Measurements

**Figure 7:**
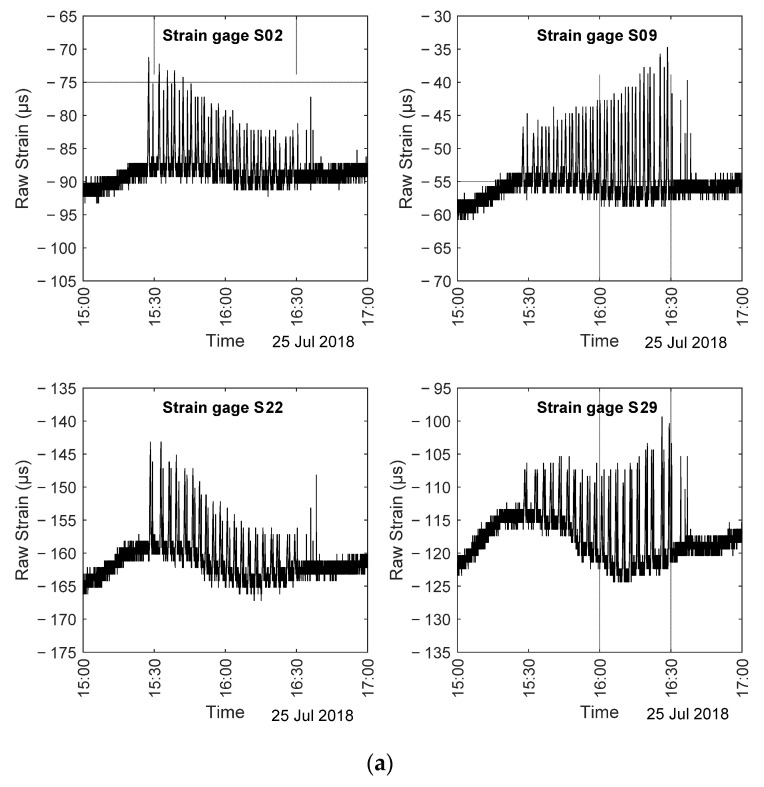

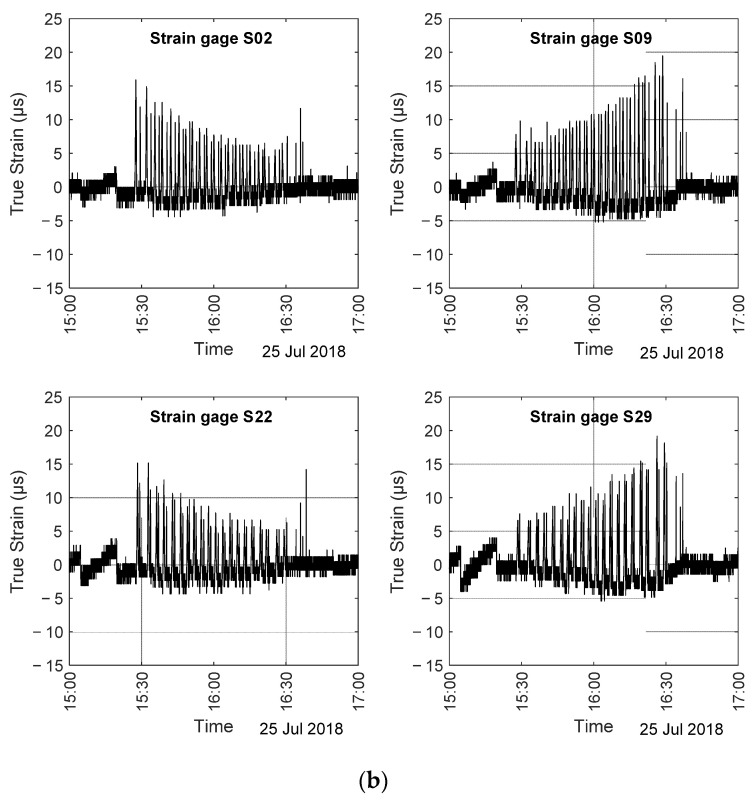
(**a**) Raw strain recorded during the truck load test; (**b**) true strain recorded during the truck load test.

The strains relative to the truck load test were downloaded from a repository with permission from the research sponsor. These values are hereinafter referred to as the “raw data”. The difference between the raw strain and the 15 min moving averaged strain was then calculated and hereinafter referred as the true strain. This difference only includes transient components of the strain and any long-term effects such as temperature-induced strains are filtered out. Please note that the term “True Strain” here is only the strain values subtracted from the moving average strain and it is not related to the concept of true strain applied in elasticity or nonlinear mechanics. The raw strains recorded by the closest gauges to the north (S02) and to the south (S09) parapet of Span 1 are presented in Figure 7a. This figure also includes the raw strains relative to gauges S22 and S29 on Span 2. Figure 7a shows that the truck load test triggered drastic rises in the raw strain values. However, these transient test-driven strains are added to the baseline strains. To facilitate an easier comparison between the truck load test strains, the corresponding true strains were calculated and depicted in Figure 7b. The true strain values are consistent with the fact that the test began closer to the north side, whereas the latest crossings were close to the south side. The plots showed about twenty double peaks and their values were in excellent agreement with the maximum strain increases, namely 16.6 µε, 20.7 µε, 16.1 µε, and 22.2 µε, independently reported for each gauge by the third party. The reliability of the “true strain” proposed here was further validated by the fact that the amplitude of those peaks increased or decreased depending upon the position of the gauges. For example, the peaks relative to S02 decreased as the test progressed. This is because the truck was crossing the bridge progressively away from that gauge. Conversely, the true strain relative to gauge 09 increased with the progress of the test.

## 5. Numerical Predictions

The high-fidelity model discussed in Section 2 was used to predict the static strain induced by the truck located at the midspan. The distance of the numerical load from the north parapet was left consistent with the field testing in order to compare numerical and experimental values. The truck load was applied on the deck of the FE model using six nodal forces according to the position of the wheels in Figure 6b. The crossings (tests) 5 and 6, 9 and 10, 15 and 16, and 19 and 20 were simulated.

**Figure 8:**
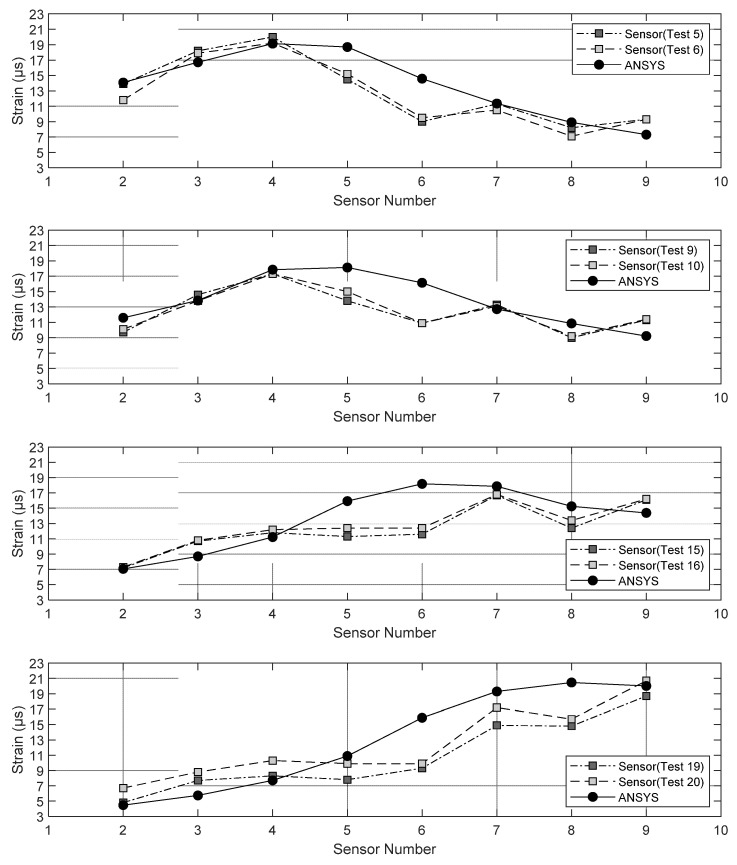
Numerical and empirical strains measured at given gauge locations when the static load (truck with certified weight) was applied to certain locations above span 1.

Figure 8 shows the predicted strains at S02 through S09 along with the empirical measurements reported independently by the third party. Owing to the field test protocol, it is noteworthy that such maximum strain may not have occurred exactly when the truck was at the location assumed in the model. Nonetheless, the numerical and the experimental values were quite consistent except for gauges 5 and 6.

**Figure 9:**
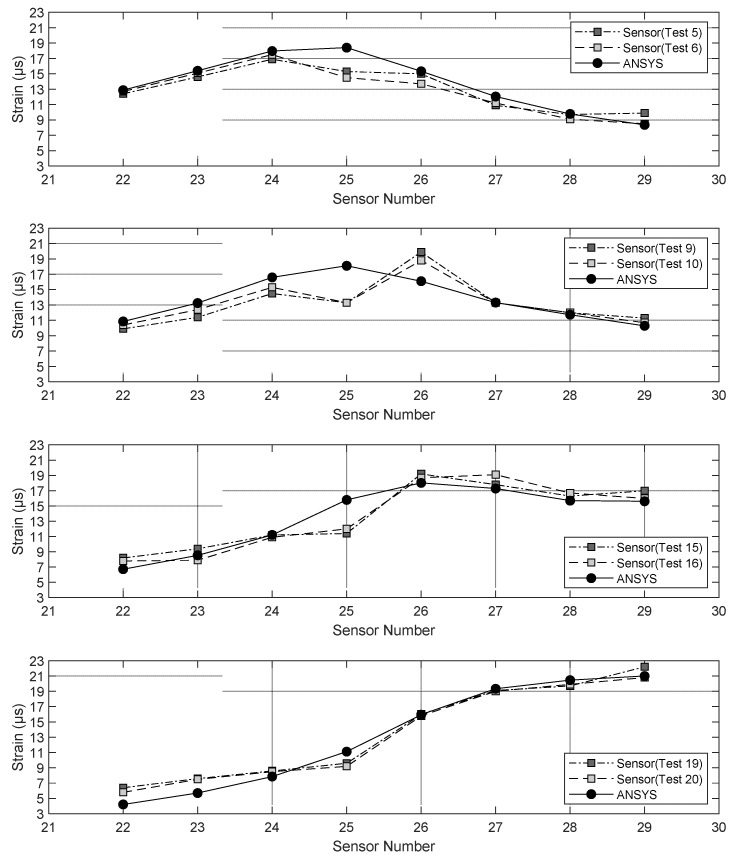
Numerical and empirical strains measured at given gauge locations when the static load (truck with certified weight) was applied to certain locations above span 2.

Similar to Figure 8, Figure 9 presents the results relative to span 2. Here, the numerical and the experimental values were even closer, with near perfect overlap for most sensors. The only mismatch was seen for gauge 25. This demonstrates the reliability of the model including the stress and strain transfer across the adjacent boxes. The fact that the readings from gauges 5, 6, and 25 mismatch the numerical estimation may be attributed to a combination of the following factors: gauge responsiveness, material properties, and local effects.

## 6. Long-Term Monitoring

**Figure 10:**
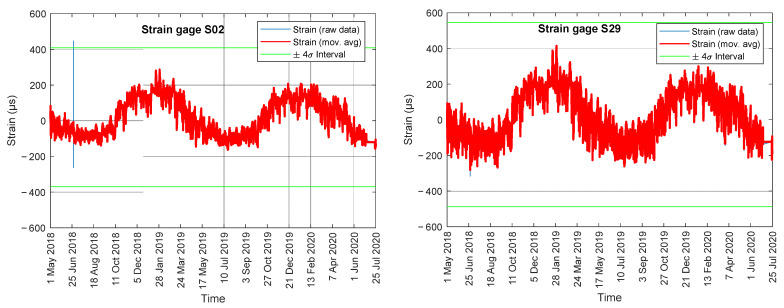
Raw strain and corresponding 15 min moving average recorded by gauges S02 and S29.

The bridge was monitored for over two years, from May 2018 to the end of July 2020. Figure 10 shows the raw strain and the corresponding 15 min strain averages for two representative gauges, namely S02 and S29, along with the ±4σ interval. There was a clear cyclic trend associated with the seasons. Most of the strains were within ± 400 µε, which is about 15-fold the strain increase experienced during the truck test. This is because the bolted-type strain gauges were not temperature-compensated. The figure shows a spike in the afternoon of 28 June 2018. The nature of this event is discussed later. Not shown here, the individual analysis of the time series such as those presented in Figure 10 showed that gauge S09 stopped working in July 2019. In addition, gauges 01, 10, 11, 20, 21, and 30 showed wider variations with respect to the other gauges. This is consistent with the fact that these gauges were located on the outer walls and therefore were more susceptible to temperature variations.

**Figure 11:**
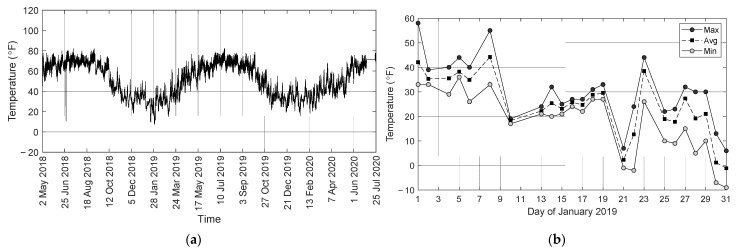
(**a**) Raw temperature recorded by gauge S02 during the long-term monitoring; (**b**) temperatures recorded by a weather station near the bridge during January 2019.

Figure 11a shows the raw temperatures recorded by the temperature gauge embedded with the strain sensor S02. The graphs present the same spike on 28 June 2018, which suggest that it was caused by some electromagnetic interference that affected the whole system. A few days of January 2019 were extremely cold as confirmed by the temperatures shown in Figure 11b recorded by a near (Johnstown-Cambria Co.) weather station (https://www.wunderground.com/history/monthly/us/pa/johnstown/KJST/date/2019-1, accessed on 10 April 2022).

**Figure 12:**
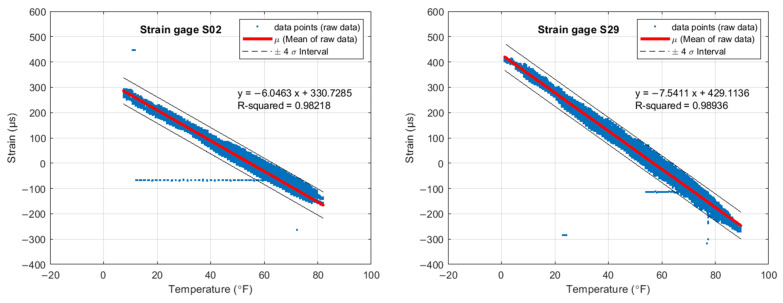
Raw strain vs. raw temperature.

Figure 12 shows the raw strain vs. the raw temperature collected for S02 and S29. Each graph contains the lines bounding the ±4σ interval and a thick red line that indicates linear regression. Instead of commonly used 3σ, here the choice of four times the standard deviations was used for better visualization of the distribution and also more effective/realistic outlier analysis. The equation of this red line and the corresponding R^2^ value are provided as well. If each individual concrete box beam was free to expand/contract without lateral confinement, the slope of the equation could have been linked to a kind of composite coefficient of linear thermal expansion.

**Figure 13:**
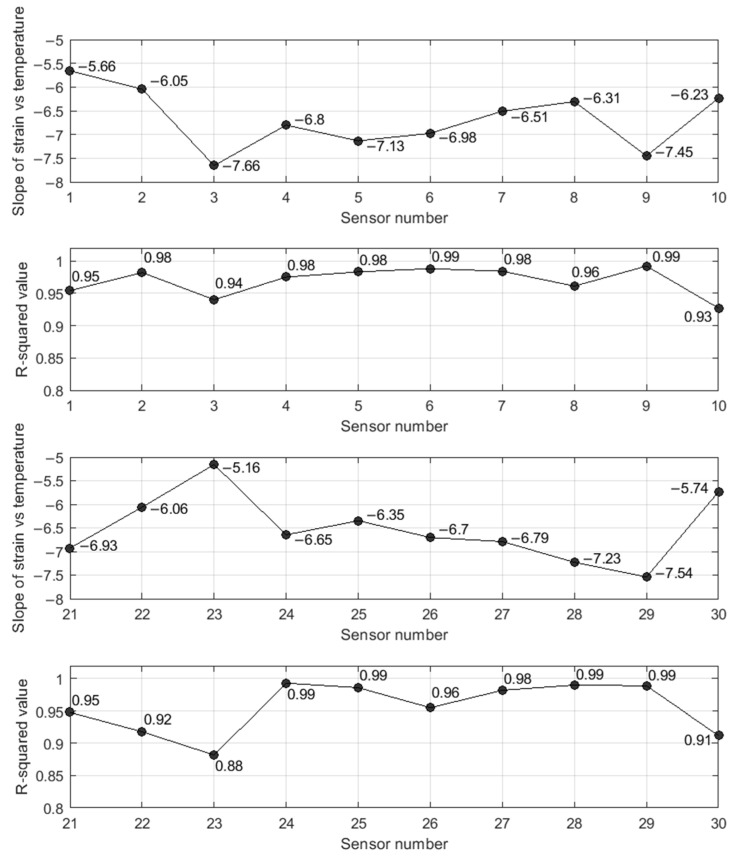
Slope of linear regression of the strain vs. temperature graphs (the values are expressed in µε/°F) and Residual R^2^ of the linear interpolation strain vs. temperature. S01–S10 were bolted to the first span and S21–S30 were bolted to the second span.

Figure 13 provides the quantitative value of the slope of the linear regression and the corresponding R^2^ associated with the sensors placed at the midspan of span 1 and span 2. As a reminder, gauges 1 and 21 were located on the exterior box on the north side of the bridge, whereas sensors 10 and 30 were located on the outer wall of the concrete box on the south side of the bridge. The following considerations can be made from Figure 13. With very few exceptions, the R^2^ values were equal to 0.98–0.99. This strong linear relationship is biased by the fact that the strain gauges were not temperature compensated. The temperature dependence of the sensors is the most dominant factor and can be used to identify anomalies that may be related to the unresponsiveness of the structural members to thermal stress. The values of R^2^ suggest that neither concrete box seemed to be “locked”, and therefore they can freely expand/contract. All the slopes were negative, i.e., negative or compressive strains develop when the temperature increases. The largest gradient, equal to −7.66 µε/°F was found for gauges 03 and 11; the smallest gradient, equal to −5.04 µε/°F was found for gage 20. The gradients may reveal bridge members that respond differently to temperature effects. Adjacent members do not display similar gradients. For example, S02, S03, and S04 may vary as much as 20%. The same can be said for gauges S22, S23, and S24. The gauges located at the midspan of span 1 did not show the same gradient pattern as their counterparts on span 2. For example, the gradient of gauge 03 was equal to −7.66 µε/°F whereas the gradient of gauge 23 was equal to −5.16 µε/°F, i.e., 30% smaller. Some of the gauges with the highest (in absolute value) gradient were close to areas of the bridge with spalling found during a previous bridge inspection. However, there is no clear evidence that such a consideration can be generalized.

Figure 12 and Figure 13 helped determining the structural response of the bridge to thermal effects and therefore to identify any long-term effects. The “true strain” was computed to identify transient events that may have overloaded the bridge. However, it was found that this approach isolated many spikes from many gauges, and these strain peaks were much higher than the maximum strain increase recorded during the truck test. After due analyses of these potential harmful events, it was found that they were false positives triggered by temporary data outage mostly caused by either the absence of traffic or isolated issues with the hardware or software.

**Figure 14:**
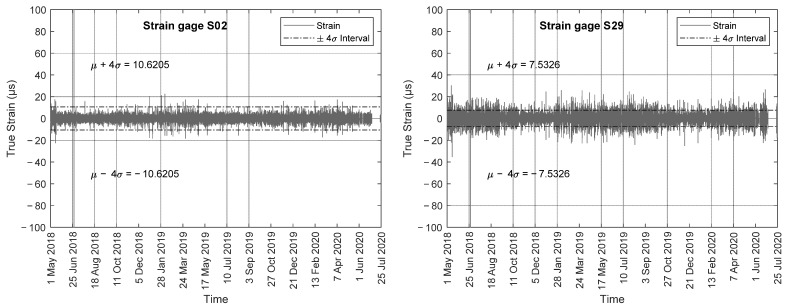
Cleansed true strain calculated as the difference between the raw strain and the moving averaged strain.

The algorithm to extract the true strains was then modified as follows. First, for each raw data sample, the difference between two consecutive timestamps was computed. If this difference was at least 15 min, then the data were discarded as they would flag a large spike. The outcome of this second step is hereinafter referred to as the cleansed true strain. This further cleaning was meant to remove spikes induced by sensor blackouts. Such blackouts created unwanted spikes in the true strains while they could not be attributed to any significant live load and thus needed be removed and cleaned. The results of the procedure are presented in Figure 14, which shows the cleansed true strain for sensors S02 and S29. The graphs show a few deformations above 20 µε. According to Figure 8 and Figure 9, this value is consistent with the crossing of a truck near the south-most and the north-most side of the bridge.

Charts as Figure 14 can reveal the presence of isolated peaks associated with heavy traffic or other sudden transient events. This is possible by comparing the values of the live loads with the value of the maximum strain recorded during the scheduled load truck test, and by setting a threshold equal to four times the standard deviation. Because the values plotted on the graphs were obtained by subtracting the raw strain from the 15 min moving average, the above graphs cannot reveal any sensor drift and they cannot detect any permanent or anomalous deformation of the structural members. These graphs are not adversely affected by the offset induced by the sensor installation. In addition, these graphs may offer a valuable tool to identify isolated peaks (outliers) that may be triggered by overloads, impacts, or other unscheduled/unplanned transient activities. Overall, it is believed that some heavy trucks crossed the bridge. However, the most notable peaks were likely related to electromagnetic interference or a temporary issue with the software/hardware.

**Figure 15:**
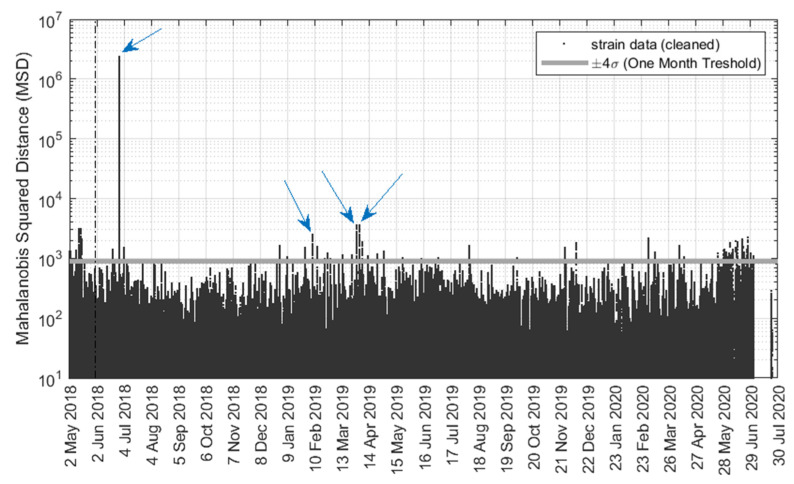
Mahalanobis squared distance applied to the live strains from all thirty gauges. Few outliers are specified with arrows.

The previous analyses were based on the examination of the individual sensors and the subsequent comparison of data from sensors that were bolted to structurally similar elements. The outlier analysis (OA) described next complemented the above analyses, in order to detect anomalies automatically and to increase the reliability of detecting anomalous events by considering the entire set of data, by-passing the check of the individual sensors. OA is a novelty detection method that establishes whether a new configuration of the system is discordant or inconsistent from the baseline configuration, which consists of an existing set of data (or patterns) that describe the normal operative conditions. In the study presented in this paper, OA was applied using the cleansed data from the twenty strain gauges along with the gauges located close to the support of span 1. The Mahalanobis squared distance (MSD), D_ζ_, a scalar defined as [40,41,42]

(1) $$D_{\zeta }={\left(\left\{x_{\zeta }\right\}-\left\{\bar{x}\right\}\right)}^{T}\cdot {\left[K\right]}^{-1}\cdot \left(\left\{x_{\zeta }\right\}-\left\{\bar{x}\right\}\right)$$

was computed. In Equation (1), {*x*_ζ_} is the potential outlier vector, $\left\{\bar{x}\right\}$ is the mean vector of the baseline, [*K*] is the covariance matrix of the baseline, and T is the transpose symbol. The MSD applied to the cleaned live load strain recorded from the ten strain gauges is presented in Figure 15. For this analysis, the data relative to the month of May 2018 were considered to be the baseline, and each datum from the rest of the year was compared to the baseline. The graph is presented in semi-log scale and a few outstanding outliers are visible. The four largest outlier spikes correspond to 28 June in 2018, 7 February, 31 March and 3 April in 2019, just to mention a few and their associated timestamps (please observe the arrows referring to these four cases in Figure 15). Interestingly, the electromagnetic interference event on 28 June observed earlier stands out in the outlier analysis as well.

**Figure 16:**
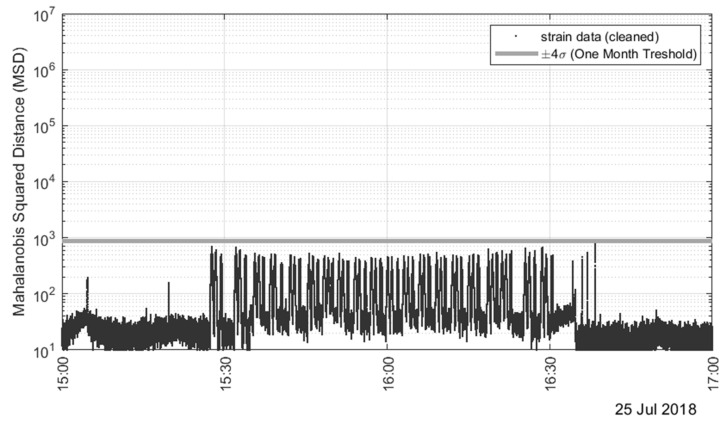
Close-up view of the Mahalanobis squared distance during the truck test.

To verify how these outliers compare to the MSD calculated for the truck test data, Figure 16 shows a close-up view of the MSD relative to the truck load test. The fact that there are only inliers suggests that the outliers seen in Figure 15 are likely due to heavy truck crossings. However, it cannot be excluded that some cases might have been related to a transient anomaly from the SHM system. For example, the largest spike in Figure 15 was observed on 28 June 2018, which has the same exact time stamp as the electromagnetic interference spikes shown in Figure 13 and Figure 14. This corroborates even further the hypothesis that this high peak is not indicative of a real transient heavy live load or physical event, but it is due to a temporary and isolated malfunctioning of the gauges or the software.

## 7. Conclusions

This paper presented a case study about the monitoring of a two-span bridge using an array of strain gauges bolted to the bridge. The field data from a controlled truckload test and from the long-term monitoring were collected from a repository and processed using simple but effective algorithms. The monitoring was complemented by and with the development of a high-fidelity model in which every element of the bridge was included in the model. The numerical prediction and the experimental values were compared, showing that the deck performed fairly well in distributing the loads to the substructure, and to the adjacent boxes. Based on the strain measurements, is it believed that both spans of the bridge were not subjected to the onset of new critical damage or the critical progression of any existing critical damage. However, it was observed that a few gauges in the first span (S5, S6, and S8) and one gauge in the second span (S25) did not respond as predicted during the truckload tests.

The study presented in this paper showed the advantages of physics-based monitoring methods with respect to data-driven SHM that can be used when the recorded data are not sufficient for data-driven methods. An ongoing study is evaluating the response of the bridge under different damage scenarios.

## Data Availability

Not applicable.
